# Cellular heterogeneity in disease severity and clinical outcome: Granular understanding of immune response is key

**DOI:** 10.3389/fimmu.2022.973070

**Published:** 2022-08-22

**Authors:** Kriti Khare, Rajesh Pandey

**Affiliations:** ^1^ Immunology and Infectious Disease Biology, INtegrative GENomics of HOst-PathogEn (INGEN-HOPE) laboratory, CSIR-Institute of Genomics and Integrative Biology (CSIR-IGIB), Delhi, India; ^2^ Academy of Scientific and Innovative Research (AcSIR), Ghaziabad, India

**Keywords:** cellular heterogeneity, infectious disease, immune response, COVID-19, RNA-seq, single-cell genomics

## Abstract

During an infectious disease progression, it is crucial to understand the cellular heterogeneity underlying the differential immune response landscape that will augment the precise information of the disease severity modulators, leading to differential clinical outcome. Patients with COVID-19 display a complex yet regulated immune profile with a heterogeneous array of clinical manifestation that delineates disease severity sub-phenotypes and worst clinical outcomes. Therefore, it is necessary to elucidate/understand/enumerate the role of cellular heterogeneity during COVID-19 disease to understand the underlying immunological mechanisms regulating the disease severity. This article aims to comprehend the current findings regarding dysregulation and impairment of immune response in COVID-19 disease severity sub-phenotypes and relate them to a wide array of heterogeneous populations of immune cells. On the basis of the findings, it suggests a possible functional correlation between cellular heterogeneity and the COVID-19 disease severity. It highlights the plausible modulators of age, gender, comorbidities, and hosts’ genetics that may be considered relevant in regulating the host response and subsequently the COVID-19 disease severity. Finally, it aims to highlight challenges in COVID-19 disease that can be achieved by the application of single-cell genomics, which may aid in delineating the heterogeneity with more granular understanding. This will augment our future pandemic preparedness with possibility to identify the subset of patients with increased diseased severity.

## Introduction

Cellular heterogeneity is defined as an inherent attribute of biological systems that contribute to genetic diversity. Nearly all the cellular systems within an organism are heterogeneous (1). However, there may be differences in the hierarchy of heterogeneity at different levels of expression or regulation. Multicellular organisms undergo specialization, allowing cells to carry out distinct physiological activities. Despite having similar or nearly identical genomic architecture, these cells differentiate in function by retaining diverse (in nature) but specific (in function) gene expression profiles throughout developmental and disease conditions. Hence, it is necessary to unravel the cellular heterogeneity occurring originally *via* a specific infection toward differentiating healthy and disease states. Therefore, understanding the diversity of disease severity and clinical outcomes, requires uncovering the functional diversity of cells. The outcome modulation by this cellular heterogeneity can imply distinct functionality necessary during a diseased state for survival conditions (2).

There is a diverse array of factors that regulate cellular heterogeneity. Variable biochemical processes, such as stochastic gene and protein expression, functional variances in cell development or cell cycle status, and tissue micro-environmental alterations, are the fundamental sources of cellular heterogeneity (3, 4). Cellular heterogeneity can also be triggered by intrinsic factors such as genetic mutations during transcription and translation, genotype mediated cell switching, or extrinsic factors such as environmental induced adaptive transformation (5, 6). This leads to cell–cell variations that promote functional heterogeneity within the cell population. The competence to extensively characterize cellular heterogeneity is critical for furthering our understanding of cell activity and disease causes. However, because of the complexity in cell diversity, it becomes a cumbersome task to identify significant and specific cellular subgroups that may be responsible for several infectious diseases.

In recent times, several high-throughput sequencing approaches have enabled study of cellular heterogeneity across infectious diseases. Next-generation sequencing (NGS) platforms have been critical toward this as they generate huge amounts of data (7). With bulk NGS analysis, millions of cells can be sequenced at once, but the understanding of distinct cell and tissue type is compromised *vis*-*à*-*vis* extent and functional role of cell heterogeneity (8). During the last few years, single-cell NGS (scNGS) has potential to alleviate limitations associated with the bulk NGS by allowing sequences to be linked to a single cell at the proteomic, epigenomic, transcriptomic, and genetic levels (9).

This article aims at highlighting the role of cellular heterogeneity across infectious disease, particularly the COVID-19. This includes the different levels of immune response defining the host’s response with disease phenotype and correlation of the heterogeneous population of immune cells with COVID-19 disease severity and clinical outcome. It provides a compendium of insights on the possible clinical outcome of a disease from the cellular heterogeneity perspective, based on the clinical phenotypes.

## Cellular heterogeneity across infectious diseases

### COVID-19 disease severity sub-phenotype

While understanding the infectious diseases, COVID-19 pandemic has surpassed the previous known global infectivity and is one of the major infectious diseases of the era. The single-stranded RNA virus, severe acute respiratory syndrome coronavirus 2 (SARS-CoV-2), causes COVID-19, which is a heterogeneous disease with a variable range of severity symptoms. The clinical presentation of COVID-19 varies from predominantly asymptomatic and mild-to-moderate episodes to more severe and critical, where 10%–20% of patients develop acute respiratory distress syndrome (ARDS) and pneumonia (10, 11). Although the viral genetic diversity, genetic evolution, variable infectivity, or co-pathogenesis contribute to infectivity and fatality, there are missing links to explain the observed diversity of disease heterogeneity for COVID-19. It seems that an important contribution toward the disease heterogeneity modulation is by the human host immune response itself (12).

The COVID-19 disease severity sub-phenotype is categorized by World Health Organization (WHO) into asymptomatic, mild, moderate, severe, and critical, where there is a clear distinction based on clinical symptoms that they exhibit (**Table 1**) (Clinical management of COVID-19: interim guidance, WHO). Most of the SARS-CoV-2–infected individuals display a mild form of disease that is generally asymptomatic, whereas a few people progress toward a severe or critical phase that necessitates intensive care unit admissions. In addition to the diverse clinical symptoms, the SARS-CoV-2–infected individuals also manifest differential immune responses. Studies have reported that most patients with severe COVID-19 have elevated plasma levels of pro-inflammatory cytokines, interleukin-6 (IL-6), and IL-1β, along with monocyte chemoattractant protein 1 (MCP-1), interferon gamma (IFN-γ)–induced protein 10 (IP-10), and granulocyte colony-stimulating factor (G-CSF) (10). Severe patients have reported elevated levels of inflammatory neutrophils and monocytes, a dramatic decrease in lymphocytes, and an inflammatory environment including IL-1, IL-6, and tumor necrosis factor (TNF) (previously known as TNFα) (13–16). Pro-inflammatory cytokines such TNF, MCP-1 (CCL2), and macrophage inflammatory protein 1a (CCL3) were found to be present at higher levels in severe cases, indicating a robust inflammatory response (10). Further research demonstrated a unique cytokine response with chemokine-enriched signature and activated IL-1 and IL-6 pathways (17, 18). Patients with severe COVID-19 compared with mild patients and healthy individuals revealed a reduced frequency of T cells, accompanied by an increased frequency of monocytes (19). The presence of a high concentration of pro-inflammatory cytokines over the course of disease in severe patients, whereas its lower concentration in patients with mild symptoms, suggests an innate signature shift between the early and late stages of the disease, leading to a divergence of patients into mild and severe COVID-19 over the disease course (19).

**Table 1:** Classification of COVID-19 disease severity sub-phenotypes.

Category	Infection status	Disease symptoms
Asymptomatic	SARS-CoV-2 +ve	no COVID-19 associated symptoms
Mild	SARS-CoV-2 +ve	fever, sore throat, malaise but no shortness of breath with SpO2 >94%
Moderate	SARS-CoV-2 +ve	clinical signs of pneumonia, breathing difficulty with SpO2 ≥ 90%
Severe	SARS-CoV-2 +ve	clinical signs of pneumonia, severe respiratory distress with SpO2 <90%
Critical	SARS-CoV-2 +ve	clinical signs of severe pneumonia, acute respiratory distress syndrome, respiratory failure, multi-organ failure

**Figure 1:**
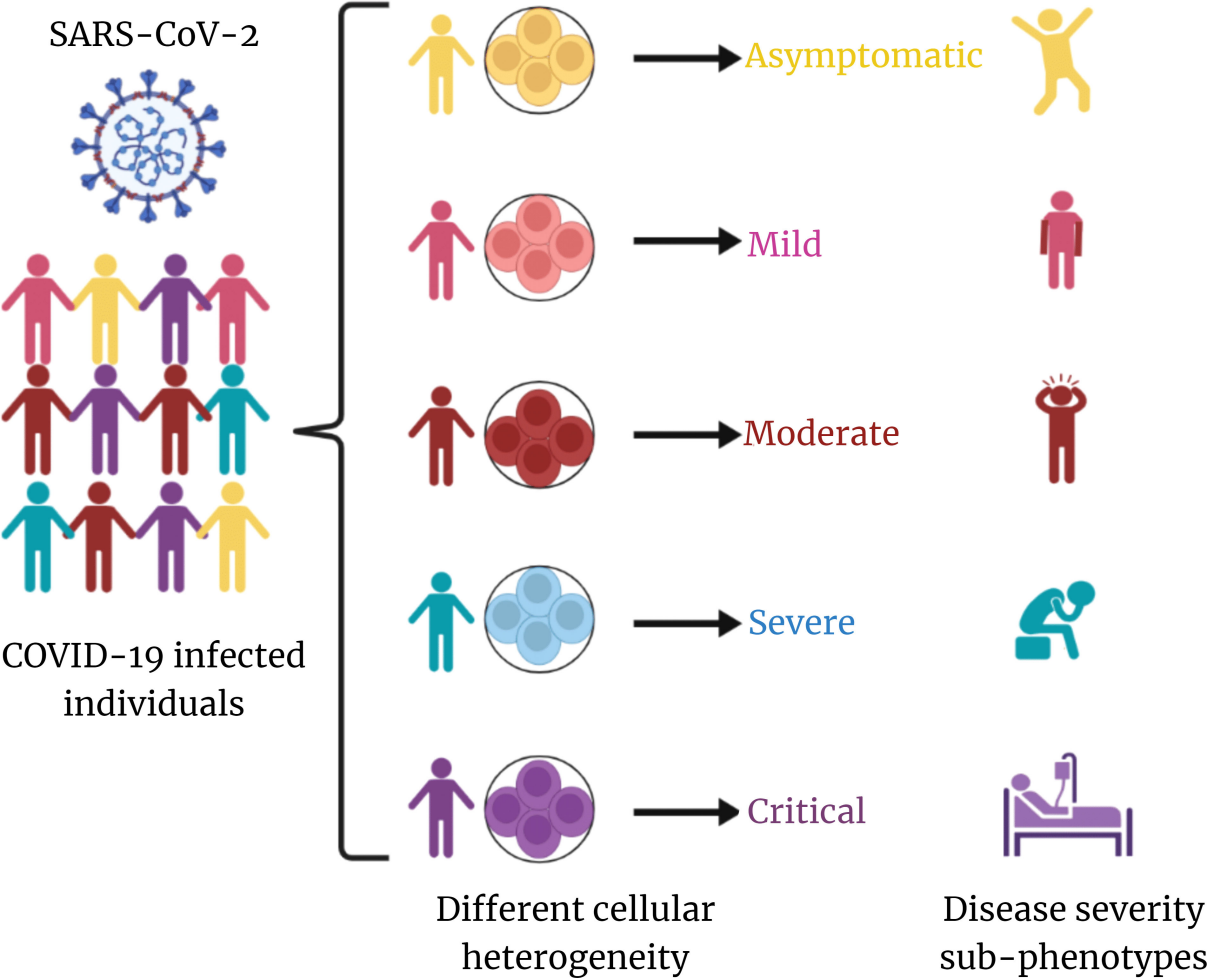
Overview of cellular heterogeneity across COVID-19 disease. SARS-CoV-2–infected individuals exhibit distinct cellular heterogeneity, which is contributing toward the diversity in COVID-19 disease severity sub-phenotypes. This is at the core of differential clinical outcome of recovered and mortality albeit infected by same/similar clade/lineage of the virus.

Although there is an increasing amount of knowledge on the host immune response to SARS-CoV-2 infection and the pathogenesis of COVID-19, it is still not apparent why some patients progress to severe illness, whereas others present mild symptoms or are asymptomatic. Therefore, it is crucial to define the immunological and inflammatory components of SARS-CoV-2 infection in great detail. The rationale behind different clinical outcomes based on immune profiles of patients infected with similar viral strain could be possibly due to cellular heterogeneity (**Figure 1**).

## Different layers of cellular heterogeneity in COVID-19

The role of cellular heterogeneity is essential to understand the mechanism by which a complex network of billions of specialized immune cells functions in harmony and produces a coordinated host response against COVID-19. During a viral infection, there are two different layers of the immune system, i.e., innate and adaptive, which functions one after the other to restrict the entry of virus or prevent it from bypassing the host’s immune system (20, 21). The innate immune system provides the first line of defense during the initial phase of viral infection, which is followed by the activation of adaptive immune response. The similar response has been observed during COVID-19 as well, where the two defense systems attempt to diminish the deleterious effects caused during and after the SARS-CoV-2 infection.

### Innate immune response

Innate immune cells carry pathogen recognition receptors (PRRs) on their surface that, upon viral entry, trigger inflammatory signals through recognition and binding of harmful viral particles that behave as pathogen-associated molecular patterns. On the other hand, intrinsic compounds, such as heat shock proteins, are released from viral-infected or damaged cells that act as damage-associated molecular patterns and activate innate immune and inflammatory responses by interacting with PRRs. Nucleotide-binding oligomerization domain, Toll-like receptors (TLRs), C-type lectin receptors, a retinoic acid–inducible gene I–like receptors (RLRs), and absent in melanoma 2–like receptors are the most prevalent PRRs in the host (22). Among these receptors, RLRs and TLRs are two crucial receptors for detecting RNA viral infection. These receptors are known to activate signaling-related pathways, such as IFN signaling, cytokine production, and cell death in response to the SARS-CoV-2 infection (23). However, SARS-CoV-2 counteracts host’s innate immune defenses by encoding proteins and mechanisms that ultimately leads to hyperactivation of the innate immune response, which is a hallmark of COVID-19 severity (24). Recent studies have revealed several mechanisms employed by SARS-CoV-2 to restrict the overall cellular antiviral state, simultaneously leading to hyperactivation of the innate immune response. This involves the following: prevention of PRRs’ sensing of viral RNA by encoding non-structural proteins; inhibition of PRR-mediated signaling pathways by encoding structural proteins (M, membrane; N, Nuclear); and viral protease-mediated cleavage by encoding two proteases (PLpro and 3Clpro), which negatively impacts the innate immune system (25–28).

The innate immune cells are the primary target for successful SARS-CoV-2 infection (29). Macrophages, monocytes, neutrophils, and natural killer (NK) cells are the major types of innate immune cells that are known to activate the downstream signaling of inflammatory response during COVID-19 (23). However, the outcome among COVID-19 disease sub-phenotypes is unique, which indicates the presence of a heterogeneous population of immune cells functioning in response to the SARS-CoV-2 infection. According to previous studies, patients with severe COVID-19 have decreased abundance of monocytes dendritic cells (DCs) and NK cells together with increased proportions of neutrophils, macrophages, and monocyte-derived suppressor cells when compared with patients with mild COVID-19 (30–35). Increase in myeloid-derived suppressor cell population is known to suppress the T-cell functions, leading to the dampening of excessive immune response and simultaneously the inflammatory phenomenon (36). This brings out the diversity within a major class of innate immune cells responding toward sub-phenotypes of COVID-19.

Furthermore, a heterogeneous understanding of innate immune cells can help in functional understanding of the clinical outcome with a dysregulated immune profile of individuals affected with SARS-CoV-2. Xu et al. observed a significant difference among the myeloid cells (monocytes and DCs) composition between patients with severe and mild COVID-19. This was supported by an increased CD14+ monocyte population and decreased CD16+ non-classical monocyte, CD14+CD16+ monocytes, and DC2 in severe patients compared with mild patients and healthy individuals (37). Whereas classical CD14+ monocyte is critical for initiating initial inflammatory response, non-classical monocytes are majorly involved in immune surveillance (38, 39). In a study, inflammatory HLA-DRhiCD11chiCD14+ monocytes were abundant in individuals with mild COVID-19 whereas the severe patients exhibited decreased HLA-DR expression (40). The expression of HLA-DR is a pro-inflammatory sign, where an increased population of inflammatory HLA-DRhiCD11chiCD14+ monocytes in patients with mild COVID-19 suggests the activation of the innate immune response, whereas its reduced expression may lead to immune suppression in patients with severe COVID-19. This decrease in monocyte subpopulations indicates a monocyte dysfunction that may be caused due to the presence of increased levels of IL-6 in patients with severe COVID-19 (41). The macrophages also revealed heterogeneity, where monocyte-derived macrophages were abundant in severe patients, thereby exhibiting hyper-inflammatory conditions (34, 42). Monocyte-derived macrophages have also been linked as a possible source of pro-inflammatory cytokines, TNF, CXCL-8, IL-1, and IL-8 during severe COVID-19, therefore suggesting a strong association between monocyte-derived macrophages and COVID-19 disease severity (37, 43). The upregulation of pro-inflammatory genes in innate immune cells of severe patients mainly belongs to the Nuclear factor kappa-light-chain-enhancer of activated B cells (NF-kB) pathway (44). Therefore, an excess of pathogenic inflammatory neutrophils and macrophages may result from the buildup of NF-kB–dependent pro-inflammatory mediators, and these cells may continue to release pro-inflammatory cytokines and chemokines, including CCL2, CCL3, CCL5, CCL8, TNF, CXCL2, CXCL8, CXCL9, CXCL16, IL-1, IL-17, IL-1RA, IFN-γ, IP-10, MCP-1, G-CSF, and GM-CSF (10, 18, 45–47). A significant increase in the immature neutrophil population, which show evidence of recent activation including increased surface expression of CD64, RANK, RANKL, PD-L1, and reduced CD62L expression, suggests a suppressive character of neutrophils in severe COVID-19 (40). NK-cell subpopulation consists of two subsets based on their relative surface expression of CD56 and CD16 receptors. The CD56^bright^CD16^neg^ NK-cell subset majorly produces cytokines, and the CD56^dim^CD16^pos^ NK-cell subset is characterized by strong cytotoxicity and high expression of inhibitory receptors such as killer cell immunoglobulin-like receptors. (48). Among these NK-cell subsets, the frequency of CD56^bright^CD16^neg^ NK cells has been reported to be depleted in severe patients, suggesting their involvement in mediating the COVID-19 disease severity (49). In contrast, reduced NK-cell subpopulation within severe revealed a reduced CD16 expression (33), which indicates increased NK effector activity and overproduction of cytokines like IFN-γ in response to IL-12, IL-15, and IL-18 stimulation (50). Zheng et al. reported decreased NK-cell subpopulation due to an increased expression of NKG2A, an inhibitory molecule, leading to functional exhaustion of NK cells and elevated cytotoxicity in patients with COVID-19 as compared with healthy (51). It can be suggested that inhibitory checkpoint receptors play a crucial role in reducing the activity of NK cells in patients with COVID-19. In summary, the innate immune response in patients with COVID-19 is divergent, where heterogeneous population of cells behaves differently across the disease sub-phenotypes, thereby contributing toward the diversity of clinical outcomes.

### Adaptive immune response

In contrast to the innate mechanism of host defense, the adaptive immune system exhibits specificity for its target antigen. Humoral and cellular immunity forms the adaptive or acquired immune response, where B and T lymphocytes, respectively, provide antigen-specific responses (52). The ability of B cells to mature into plasma cells, which produce a large antibody repertoire to defend against a viral pathogen, as well as the development of immunological memory to prevent recurring infections with the characterization of lymphocytes’ roles, occurs during a viral infection (53). Naïve B cells, mature lymphocytes, memory B cells, transitional B cells, and antibody-secreting plasmablasts/plasma cells are among the several circulating human B cells, each having its own phenotypic and functional subgroups. On the other hand, T lymphocytes, upon viral entry, release mainly CD4+ T cells (helper T cells) and CD8+ T cells (cytotoxic T cells), leading to a combined antiviral immune response (54, 55).

Su et al. reported that, during COVID-19, several B-cell subsets, including naive B cells and antibody secreting cells, were elevated in severe patients, whereas memory B cells were increased in mild patients, suggesting a heterogeneous B-cell population across COVID-19 disease sub-phenotypes (55). Patients with severe COVID-19 exhibited expansion of plasmablasts along with elevated levels of SARS-CoV-2 spike receptor binding domain (RBD)–specific IgM and IgG compared with the healthy individuals, indicating an altered B-cell subset with a strong SARS-CoV-2–specific humoral response (33). Among memory B cells, the frequency of both class-switched and non–class-switched memory B cells is found to be significantly reduced in severe patients, whereas elevated expression of plasmablasts suggests that the decline in the memory B-cell population might be due to the activation of pre-existing memory cells (coronavirus other than SARS-CoV-2), further differentiating into “atypical” cells (56, 57). An increase of memory B cells in patients with mild COVID-19 demonstrates an effective and protective antibody response against SARS-CoV-2 (58). We can say that the memory B-cell subsets are negatively correlated with COVID-19 disease severity. Similarly, transitional B cells were also observed to decrease with disease severity and, therefore, display a loss of immune-suppressive regulatory B cells with an expansion of effector B-cell subsets (59). Another study also reported elevated plasmablasts, enriched T-bet+ B-cell subset, and decreased memory B-cell subsets in severe patients (60). An immunopathologic function for circulating antibody-secreting cells in severe COVID-19 is suggested by the fact that severe patients, compared with mild patients, showed increased levels of antibody-secreting cells (61). Together, these findings indicate that the dynamic B-cell heterogeneity might be modulating COVID-19 disease toward severity.

**Figure 2:**
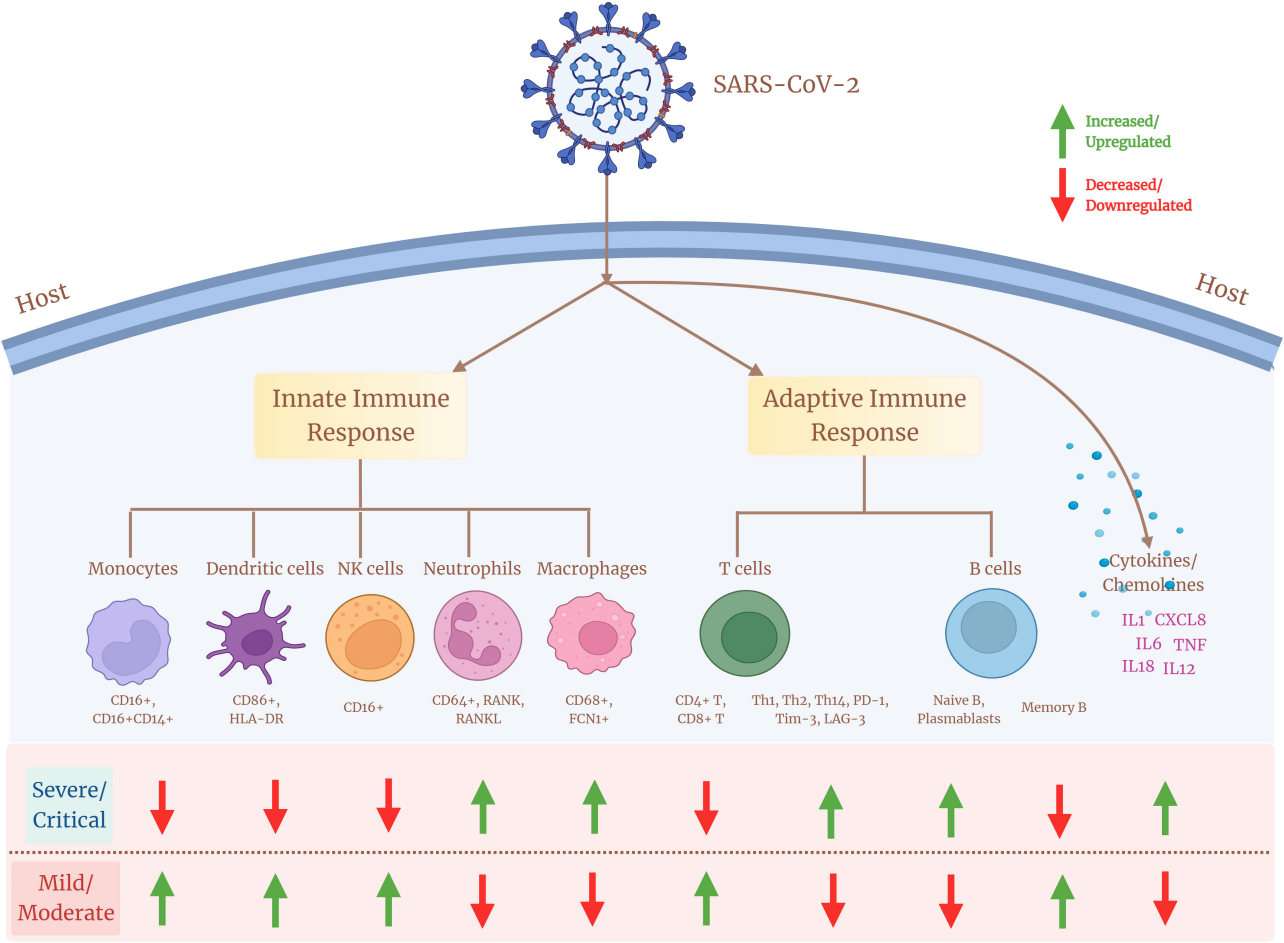
Cellular heterogeneity across the immune landscape of COVID-19 sub-disease severity phenotypes. Different cell subsets of the innate and adaptive immune system and their associated marker expression (upregulation/downregulation) showing diverse heterogeneity across patients with mild/moderate and severe/critical COVID-19.

T lymphopenia has been reported in patients with COVID-19 through multiple studies. Both CD4+ T-cell and CD8+ T-cell populations have been shown to be reduced in severe patients compared with moderate or mild patients (62). There is a negative correlation between the CD4+ T cells and COVID-19 severity, where the extended absence of SARS-CoV-2–specific CD4+ T cells is associated with severe or critical COVID-19 (63). CD4+ T-helper cell subsets also display heterogeneity across COVID-19 disease severity sub-phenotypes, where critical patients report decreased proportion of T-helper 1 (Th1) and T-helper 17 (Th17) lymphocytes, whereas T-helper 2 (Th2) cells’ percentage increased in comparison with less severe individuals (64). It leads to a lower Th1/Th2 ratio that indicates a dysregulated balance of T-helper lymphocytes, wherein decreased Th1/Th2 cell ratio and Th17 cells suggest a reduced humoral response and poor outcomes in COVID-19 disease (65). As Th1 response is essential for viral clearance, lowering of this response and increase of Th2 plausibly indicates an abnormal cellular immune response in patients with severe COVID-19. CD4+ T-cell populations are also associated with the elevated expression of exhaustive markers such as Tim-3, PD-1, and LAG-3, which aids in the progression of disease severity (66). This indicates a link toward discordant CD4+ T-cell responses, which is a likely key element in the development of severe COVID-19. An altered T-cell differentiation and cytotoxicity has been identified by Cervantes et al., wherein circulating cytotoxic CD8+ T cells have been found in higher percentage in severe patients compared with the mild patients (33). Although the absolute number of CD8+ T cells decreases in patients with severe COVID-19, the increased proportion of granzyme B and perforin produced *via* effector CD8+ T cells provides evidence of elevated cytotoxicity induced in severe patients (67, 68). Furthermore, a pronounced expression of inhibitory markers such as PD-1, Tim-3, and CD39 on CD8+ T cells in severe patients demonstrates an exhausted CD8+ T-cell population that may lead to uncontrolled cytotoxicity response, reduced cellular immunity, and tissue damage with COVID-19 disease severity (69–71). On the other hand, patients in their initial stage show a robust cellular immune response, where lower cytotoxicity and reduced exhaustive marker expression are observed, thereby suggesting a crucial role of overexpressed exhausted T-cell subpopulation in worsening COVID-19 disease severity (72). An association between hyperactivated antigen-specific T cells and COVID-19 disease pathogenesis is supported by the presence of SARS-CoV-2–specific CD8+ T cells in severe patients exhibiting elevated expression of cytotoxic and inflammatory genes, as well as greater levels of TCR clonal expansion (73). In contrast, a reduced cytokine production *via* CD8+ T-cell population in severe patients is also known. Similar to CD4+ T cells, there is evidence of functional disbalance due to increased expression of exhaustive markers by CD8+ T-cell population in patients with COVID-19 (71). CD8+ T cells from patients with severe COVID-19 produced less cytokine when stimulated (51). The study also suggests that CD8+ T cells exhibit a hyperactivation profile with enhanced cytotoxicity (33). These findings provide evidence for heterogeneous and diverse patterns of CD8+ T-cell responses across patients with COVID-19. Therefore, it can be concluded that, even in patients with severe COVID-19, a significant heterogeneity occurs due to differences in the expression of the T-cell population, leading to diverse clinical outcomes. Together, COVID-19 disease demonstrates a major impact on the innate and adaptive immune system (**Table 2**), where the response is a variable, thus reflecting a diversified outcome across the infected population (**Figure 2**).

**Table 2:** Major impact on the innate and adaptive immune response during COVID-19 infection.

Immune response	Observations during COVID-19 (compared to mild/moderate)	References
**Innate**		
Monocytes	An overall reduced abundance of monocytes in severe with increased CD14+ and decreased CD16+, CD16+CD14+ monocytes in severe COVID-19.	(37)
Dendritic cells	Decrease populations of dendritic cells in severe COVID-19 with impaired CD86 and HLA-DR.	(74)
NK cells	Decreased absolute number of circulating NK cells with elevated levels of pro-inflammatory cytokines in severe COVID-19.	(75)
Neutrophils	Increased abundance of neutrophils in severe COVID-19 with elevated expression of pro-inflammatory cytokines and chemokines.	(33)
Macrophages	Increased proportion of macrophage, especially monocyte-derived macrophage in severe COVID-19.	(34)
Cytokines/chemokines/interferons	Increased plasma levels of pro-inflammatory cytokines and chemokines (especially IL-2, IL-6, IL-10, and TNF-α) with impaired IFN-I activation in severe COVID-19.	(46) (44)
**Adaptive**		
T cells	Lymphocytopenia and modulation in lymphocyte balance associated with a decrease in levels of CD4+ cells, CD8+ cells, Th1, and Th2 cells and increased circulating CD4+ T cells, CD8+ T cells, Th2, PD-1, Tim-3, and LAG-3 in severe COVID-19.	(76)
B cells	Elevated B cell (naive B cells and plasmablasts) population with reduced memory B cells in severe COVID-19.	(58)

## Correlation between disease severity and immune cell heterogeneity

SARS-CoV-2 has been associated with dynamic immune response, which seems to be at the root of the differential disease severity and clinical outcomes in the infected individuals globally. The presence of immune cell heterogeneity, both innate and adaptive response, during COVID-19 indicates a strong link with the disease severity, which is observed extensively. The COVID-19 severity has been correlated with several granular parameters of immature neutrophils, dysregulated myeloid cell compartments, and increased proliferative CD4+ and CD8+ T cells with heightened cytokine storm (77, 78). With increase in COVID-19 severity, the CD8+ T effector and central memory cells are compromised for their clonotype expansion (79). This suggests an overall decrease in the lymphocyte population with increasing disease severity. However, higher expression of CD8+ T cells is supported by activation of exhaustive markers, which is elevated with the progression of the disease, indicating a SARS-CoV-2–driven T-cell exhaustion in severe patients (69).

Apart from the cell mediated immune response, the correlation between COVID-19 and humoral immunity is also diverse, where higher antibody titers are associated with severe clinical manifestations. Elevated levels of anti-N IgG and IgM antibodies contribute toward the disease severity among patients with fatal COVID-19 (80). Tan et al. reported higher viral clearance rate by weak responders of IgG, whereas strong responders of IgG had lower viral clearance, thereby suggesting that a stronger antibody response supports delayed viral clearance and severity (81). It is also shown that patients with mild to moderate COVID-19 exhibit a rapid decline in the SARS-CoV-2–specific antibody titer and thus indicate a correlation between longevity of the antibody responses with disease severity (82, 83). The presence of increased anti-spike and anti-RBD antibody levels with elevated expression of pro-inflammatory markers in severe patients indicates a correlation between elevated antibody response and inflammation (84). Although increased expression levels of several cytokines and chemokines mediated *via* monocytes, macrophages, DCs, NK cells, or neutrophils have been observed within the severe patients (13, 18), only a few among them such as CRP, CXCL8, CCL20, IL-6, IL-8, IL-10, PTX3, MCP-3, and IP-10 (85–88) serve as “biomarkers” and indicate a “cytokine storm” underlying COVID-19 severity. Hence, the wide range of impact of COVID-19 on the immune cell population explains the heterogeneous complexity underlying the seriousness of infectious disease and its correlation with disease severity.

## Possible modulators of COVID-19 severity

### Effect of age

Aging generates significant biochemical changes in the immune system, which have been linked to age-related disorders and infectious disease vulnerability (89–91). The impact of COVID-19 is associated with age, where the severity is observed in elderly patients compared with young adults (92, 93). Studies show that older patients with COVID-19 had higher rates of lymphopenia and the elevation of inflammatory markers such as IL-6 and CRP, which are significantly correlated with disease severity (94). Age-related imbalances in immune response and cellular activity tend to reduce innate activation of a previously weakened adaptive immune system. This chronic long-term stimulation of the innate with a reduced adaptive immune system accentuates aged people toward infection/s. Therefore, the older population has been more susceptible toward COVID-19 and exhibits a diverse immune profile (**Table 3**). On the other hand, children are less susceptible to becoming SARS-CoV-2–infected, which might be due to cross-immune protection from other coronaviruses or non-specific protection induced by other respiratory viruses (101–103).

**Table 3:** Effect of age on the immune landscape of COVID-19–affected individuals.

Immune response	Key observations in COVID-19–affected older age group (compared to younger)	Reference
**Innate**		
Monocytes	Reduced antigen presentation with accumulation of non-classical monocytes and downregulated HLA-DR.	(95)
Macrophage	Imbalance between pro-inflammatory and pro-repair macrophages.	(96)
Dendritic cells	Impaired antigen presentation with reduced CD40 and CD80; alterations in DC maturation affects T-cell activation.	(97)
NK cells	Depleted and dysfunctional NK cell with reduced cytotoxicity.	(96)
Neutrophils	Increased neutrophil count with high neutrophil to lymphocyte ratio.	(98)
Inflammatory response	Delayed type I IFN response, heightened activation of NLRP3 inflammasome with IL-1β and IL-18 levels.	(99)
**Adaptive**		
T cells	Rapid activation of CD8+ T cells, loss of anti-inflammatory Treg suppression, increased Th1/Th2 ratio, and Th17 expression leading to cytokine storm.	(100) (96)
B cells	Decreased antibody affinity, reduced production of naive B cells, and increased tissue-specific antibody-experienced memory cell.	(100)

### Effect of gender

Worldwide, greater COVID-19 mortality rates have been reported in men than in women (104), suggesting that men may be more susceptible to COVID-19 and progress toward severity. This can be correlated with the presence of stronger innate immune responses in women than in men, which possibly allows for faster viral detection and production of interferon and inflammatory cytokines, leading to faster viral clearance (105). X-chromosome harbors many PRRs genes (TLR-7 and 8), ACE2, and interleukins that confer its association with innate and adaptive immunity. A study highlighted that, compared with women, men lack the extra X-chromosome and are therefore more susceptible toward COVID-19 severity (106). TLRs are also different across both genders, where men have higher TLR-2 and TLR-4 expression, whereas women have higher expression of TLR-3, TLR-7, and TLR-9 (107). Because TLR-4 has a higher binding affinity for S-protein of SARS-CoV-2 (108) and induces cytokine production, it causes severe infection in men. On the other hand, TLR-7 stimulates B cells to increase antibodies and also the production of type I IFN during viral infection, which suggests a better initial response and viral attenuation in women upon SARS-CoV-2 infection (109, 110). Macrophages, DCs, T cells, B cells, and NK cells are among the immune-related cells that express estrogen receptors (ER-alpha and ER-beta), suggesting that the female sex steroid hormone, estrogen, regulates immune-related cells to a certain extent. According to Zafari Zangeneh et al., estrogen or estradiol may play a role in regulating the pro-inflammatory immune response against SARS-CoV-2 infection and thereby increasing the anti-inflammatory and antiviral response (110).

In contrast to estrogen, testosterone has a complex role that makes men more susceptible to COVID-19. Testosterone induces ACE2 expression that further increases TMPRSS2 expression (111). Therefore, higher testosterone levels might be associated with increased SARS-CoV-2 entry and disease severity. High levels of testosterone may also lead to COVID-19 severity in men by increasing neutrophil counts and cytokine production (IL-1, IL-10, and IL-2), changing Transforming growth factor beta (TGF-β) production by immune cells, and lowering antibody production that can possibly induce cytokine storm (112). Contrarily, low testosterone levels are correlated with worse disease outcome and increased production of inflammatory markers in men during COVID-19 (113). Testosterone levels are also linked to T-cell immunological activation and have a significant correlation with CD28 expression (114), therefore indicating that low testosterone levels may hamper the activation of the immune system during SARS-CoV-2 infection. This indicates that testosterone acts as a dual edge sword in modulating the COVID-19 severity and further elucidation would help infer the association between testosterone and COVID-19.

### Effect of comorbidities

COVID-19 is also associated with several comorbidities that play crucial roles in modulating the disease severity. According to Zhou et al., almost 50% of patients with COVID-19 in their study had either hypertension, diabetes, or coronary heart disease (115). Another study also reported that hypertension, cardiovascular disease, and diabetes are frequently associated comorbidities in severe patients, which correlates with poor clinical outcomes (116). However, other comorbidities such as cerebrovascular disease, chronic kidney disease (CKD), and other renal diseases have also been associated with severity and mortality in patients with COVID-19 (117). This indicates an overall systemic disruption induced by SARS-CoV-2 infection, where multiple organs apart from the site of infection are affected and responsible for COVID-19 severity. Patients with comorbid COVID-19 were also reported to have an altered immune profile compared with the non-comorbid. Del Valle et al. observed the presence of elevated levels of TNF-α and IL-8 in patients with diabetes, hypertension, and CKD-associated COVID-19, suggesting an elevated pro-inflammatory cytokine in patients having comorbidities (118). Higher expression of IL-6 and CRP with increased oxidative stress has also been observed in patients having diabetes as an underlying comorbidity in COVID-19 (119). Together, these findings suggest a hyperactivation and elevated immune and inflammatory response during COVID-19, having at least one comorbidity that often leads to disease severity.

### Effect of genetic factors

Host genetic factors are essential determinant of an infectious disease’s susceptibility and severity. During COVID-19, compared with SARS-CoV-2 virus, the host’s genetic variants have an important contribution toward the progression of the disease severity. Multiple genetic variants, together with candidate causal genes, are known to be associated with SARS-CoV-2 infection susceptibility and COVID-19 severity (**Table 4**). *SLC6A20*, one of the genes among the cluster of six genes (*SLC6A20*, *CCR9*, *CXCR6*, *FYCO1*, and *LZTFL1*) is present on the 3p21.31 locus and encodes the sodium–imino acid (proline) transporter 1 (SIT1), which is known to interact with the ACE2 receptor (128) and therefore may facilitate the entry of SARS-CoV-2. In particular, the Single nucleotide polymorphism (SNP) rs11385942 is present on the chromosome 3 at 3p21.31 locus and spans the six gene-containing clusters and has been associated with respiratory failure in patients with COVID-19 (120). Hospitalization of patients with COVID-19 and the severity of the disease are associated with a different mutation, rs1886814, in the transcription factor *FOXP4* (129). A candidate causal variant rs10774671, present in the splice region of *OAS1*, has been linked with COVID-19 severity (130). *DPP9* is a serine protease encoding gene that is involved in antigen presentation, inflammasome activation, and antiviral signaling (131). Its variant rs2109069 present at 19p13.3 locus is related to pulmonary fibrosis along with COVID-19 severity (132). Blood group A individuals are observed at increased risk, whereas O blood group persons have a protective phenotype. The ABO blood group locus contains an overlapping locus 9q34.2 and identified as susceptibility loci for severe COVID-19 (133).

**Table 4:** Reported COVID-19 association with genetic variants and their respective outcomes.

S. No.	Gene/s	Variants/polymorphism	Causal genes	Associated with COVID-19 phenotype	Reference
1.	*SLC6A20*, *CCR9*, *CXCR6*, *FYCO1*, *LZTFL1*	rs11385942	*SLC6A20*	Respiratory failure	(120)
2.	*FOXP4*	rs1886814	*FOXP4*	Disease severity	(121)
3.	*HLA*	HLA-A*11:01, -B*51:01, and -C*14:02	*HLA*	Disease severity	(122)
4.	*ACE2*	rs2285666	*ACE2*	Disease severity	(123)
5.	*TMPRSS2*	rs12329760	*TMPRSS2*	Disease susceptibility	(124)
6.	*TLR7*	Rare deleterious variant	*TLR7*	Disease severity	(125)
7.	*ABO*	rs912805253	*ABO*	Disease susceptibility	(126)
8.	*DPP9*	rs2109069	*DPP9*	Disease severity	(127)
9.	*TYK2*	rs74956615	*TYK2*	Disease severity	(127)
10.	*OAS1*	rs10774671	*OAS1*	Disease severity	(127)

**Figure 3:**
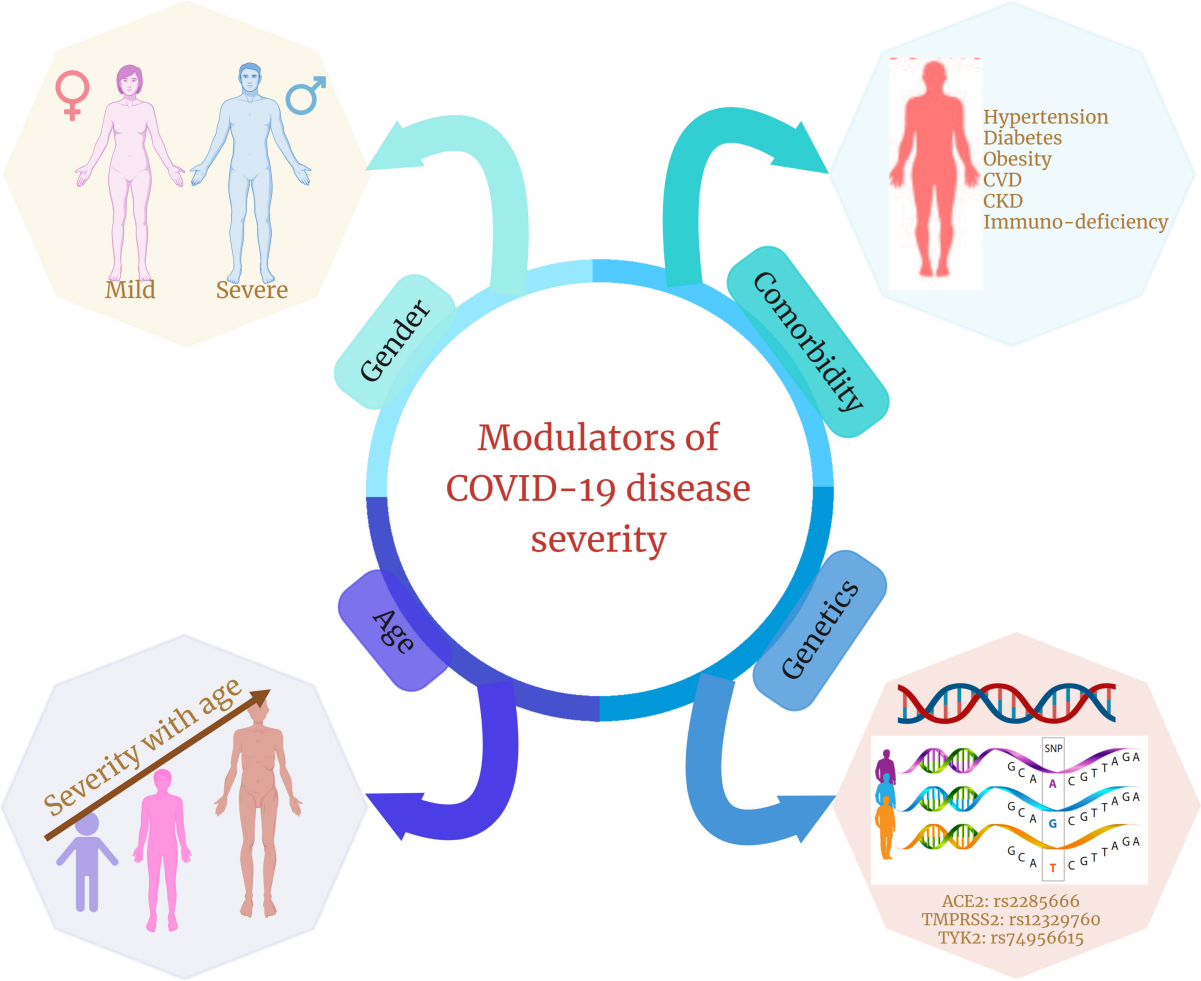
Different modulators of COVID-19 disease severity. Age, gender, presence of comorbidities, and genetic factors are four significant factors that are shown in multiple studies to modulate or are associated with COVID-19 disease severity and clinical outcomes.

IL-12, IL-23, and IFN signal transduction, as well as Th1/Th17 cell–dependent immune responses, are determined by the Janus kinase encoding gene TYK2  (134). *TYK2* variant rs74956615 has been correlated with severity of COVID-19, where high *TYK2* expression is associated with critically ill and hospitalized patients (132). *TLR7* is a potent innate immune sensor capable of recognizing viral antigen and immediately inducing interferon and other pro-inflammatory cytokines as an antiviral immune response (135). An X-linked deleterious variant of *TLR7* is reported to cause loss of function in male patients with COVID-19, which resulted in low levels of type I IFN by plasmacytoid DCs, leading to an impairment of type I interferon in response to SARS-CoV-2 infection, demonstrating the importance of functional *TLR7* in regulating the progression and severity of COVID-19 (136). *HLA* genes (both HLA-I and HLA-II) are one of the major players involved in antigen presentation to the lymphocytes, further activating the host immune response (137). There have been multiple variants of *HLA* associated with the COVID-19, among which class I HLA, including HLA-A*11:01, -B*51:01, and -C*14:02, is the significantly prevalent one associated with the worst outcome in patients with COVID-19, indicating the immuno-protective role of HLA in regulating the disease severity (122). In direct consequence toward SARS-CoV-2 infection, two major gene loci are necessary: *ACE2* and *TMPRSS2*. Huo et al. reported an association between *ACE2* and *TMPRSS2* with genetic susceptibility and COVID-19 disease severity, respectively (138). In SARS-CoV-2–infected individuals, both the alternate allele of rs2285666 for *ACE2* gene and the SNP (rs12329760) of *TMPRSS2* polymorphisms may serve as predictive model for COVID-19 severity (139). Therefore, we can say that, during COVID-19, the host’s genetic factors and the associated polymorphisms do play a significant role in determining the state of infection and disease severity (**Figure 3**).

## Discussion

Cellular heterogeneity has always been at the core for defining spectrum of infectious diseases that can be harnessed to understand the complexity underlying the disease progression. COVID-19 has been a disease of concern that has alarmed the human population globally. This heterogeneous infectious disease has affected a wide range of populations experiencing a diverse array of symptoms. The disparities between the clinical presentation and disease pattern across patients with COVID-19 are possibly due to the presence of immune cell heterogeneity (60). Severe patients reveal a completely different immune profile compared with the ones with mild clinical features. For example, severe patients compared with mild patients exhibit lymphopenia and increased monocyte population with elevated pro-inflammatory cytokines and chemokines (140). This indicates that, during COVID-19, infected individuals experience a major difference in their immune profile that regulates disease progression and affects the host’s survival.

Two levels of the defense program are activated in response to SARS-CoV-2 entry, where each endeavors to avoid any further damage to the host (141). The viral infection triggers the innate and adaptive immune responses, where different cells are programmed to perform their specific function/s in time and space. As the initial line of defense, innate immune cells, such as macrophages, DCs, monocytes, neutrophils, and NK cells, are followed by T and B lymphocytes in the adaptive immune response. Normally, the immune cells are affected during the initial stages of the disease that, with time, increases to limit any further infection. However, this trend is not always followed linearly during COVID-19. The severe patients have decreased abundance of monocytes, NK cells, and DCs, compared with mild patients (142, 143). This indicates a dysregulated myeloid cell compartment during severe COVID-19, which correlates with disease severity and ARDS development (144). However, elevated macrophage and neutrophil cell populations have been associated with overproduction of pro-inflammatory markers—CXCL8, CCL20, IFN-γ, IL-6, IL-1β, and TNF-α—across severe patients (145). These differences within innate immune cells across infected individuals indicate their abnormal expression triggered due to COVID-19, with expected major impact on the cellular landscape. These findings provide crucial information about the presence/absence of specific cell types across infected individuals based on specific markers expressed by these cells. Although there are multiple reports highlighting the upregulation/downregulation of these cell-specific markers, the exact mechanism of these heterogeneous cell populations modulating the immune response during COVID-19 is yet to be known.

Lymphopenia is a hallmark of severe COVID-19, where T- and B-cell populations are decreased with disease severity (146, 147). However, patients reveal cellular heterogeneity, especially within T-cell subsets, where the cell-mediated response is highly diverse within infected individuals. Although severe patients exhibit reduced T-cell population, they also express elevated expression of exhausted markers within T-cell subsets (both CD8+ and CD4+ T cells), suggesting a possible abnormal T-cell response throughout COVID-19 (69, 71). These heterogeneous populations of cells with an abnormal expression within patients with COVID-19 reveal a strong impact of the disease on the immune profile that provides inter-individual differences. We can say that, with disease severity, the heterogeneous immune-cell population is also altered and so is necessary to be taken forward for their functional role in the future studies.

Cellular heterogeneity also defines specific factors that can be considered as possible modulators for COVID-19 disease severity. Aging has always been a concerning issue where individuals fall into the immunocompromised category. These individuals’ immune profile is suboptimal for maintaining a healthy state among elderly, which becomes a major accelerator in COVID-19 severity. Compared with adults, the immune landscape of older individuals exhibits dysregulated innate and adaptive immune cell compartments during COVID-19. This can be correlated with inflammaging and immunoscenesce that are the defining features of infected elderly individuals and therefore indicate the observed COVID-19 severity (148–150). In addition to age, gender is also known to be associated with COVID-19 disease severity. Men, according to studies, are more vulnerable to SARS-CoV-2 infection than women (151). The presence of an extra X chromosome in women and its association with innate and adaptive immune response makes them more protective against COVID-19. Moreover, the immune cell heterogeneity is more pronounced in men as highlighted by increased neutrophil counts, heightened inflammatory response, and reduced antibody production (151, 152). Further understanding of gender-specific immune profiles is necessary to strengthen our understanding of COVID-19 with cellular heterogeneity, where gender may have a major impact on the modulation of disease severity. Specific comorbidities such as hypertension, diabetes, CKD, and cardiovascular disease correlate with higher morbidity and mortality rates in patients with COVID-19 (153). Elevated cytokines and chemokines are observed in comorbid patients compared with non-comorbid, suggesting a major alteration of the immune landscape in patients with comorbid COVID-19 (60, 119). Host’s genetic factors are of immense importance in modulating the COVID-19 disease severity. Numerous genetic variants have been studied to be associated with COVID-19 susceptibility, severity, and clinical outcomes. Among all, the specific variants associated with *HLA*, *ACE2*, and *TMPRSS2* have significant contribution in modulating the COVID-19 severity (154). A possible explanation for the effect of an HLA allele on disease severity might be abnormal binding affinity with SARS-CoV-2 peptides (155). The HLA-restricted T-cell response mechanism, in which viral epitopes are delivered by DCs to CD8+ T lymphocytes through contact with HLA class I alleles and CD4+ T lymphocytes through interactions with HLA class II alleles, is critical to the human protection mechanism. This interaction is affected in SARS-CoV-2 infection, where HLA variants have lower binding sites for SARS-CoV-2 peptides, leading to a decreased immune response and increased disease severity (156). Moreover, *TLR7-*, *DPP9-*, ABO-, *OAS1-*, and *TYK2*-associated genetic variants have been correlated with reduced antigen presentation, delayed type I IFN, and heightened cell-mediated immune response in severe patients (18, 157, 158). The respective evidence highlights the relevance of cytokine storm in COVID-19 severity and several complications, including a fatal outcome. Therefore, we can say that identifying genetic factors could aid in explaining the uncontrolled inflammatory response and, if possible, plausible biomarkers defining the COVID-19 severity that can be harnessed for therapeutic strategies.

## Challenges and future prospects

An insufficient understanding of cellular heterogeneity within patients with COVID-19 can have considerable implications for prognosis and therapeutic interventions. The disparities in clinical outcomes among COVID-19 individuals have been treated based on the supportive care and close monitoring with treatment intensification for the individuals with worsened symptoms. Hence, more stratified strategies should be opted to identify the patients with the future worse symptoms. It is necessary to dig deeper to understand patient heterogeneity that assists clinicians in utilizing intensive therapy for stratified patients and aiding potentially beneficial treatments required by an individual patient.

Immunological investigations are emerging to reveal insights into patient heterogeneity and clinical variability in patients with COVID-19 that may help understanding the disease trajectories and progression. Both B- and T-cell responses are known to be associated with the COVID-19 disease spectrum. However, the T-cell immune response has widely been shown to have a diversity among infected individuals, highlighting its significance in understanding the disease progression and patient stratification. Vital information on the involvement of T cells for COVID-19 protection is considered necessary and presently awaiting functional elucidation. Longitudinal studies of patients with COVID-19 may help to overcome this limitation by providing a precise understanding of heterogeneity within T cells and T-cell–mediated immune response across patients with COVID-19. It would be crucial to characterize the protective T-cell responses following infection and vaccination. In doing this, the single-cell genomics approach can be helpful in delineating the complex immune profile of infected individuals. Single-cell RNA sequencing (scRNA-seq) is of paramount importance in providing relevant information at single-cell resolution with the highest granularity that can aid in a comprehensive understanding of cellular immune response during COVID-19. Rather than using composites or averages from multiple cells, single-cell approaches offer a high-resolution perspective into these subsets and individual cells. Furthermore, multi-omics profiling of concurrent readouts from the same cell, including gene expression, cell surface proteins, and receptor sequences, enables more accurate and reliable characterization of these heterogeneous cell populations. From evidence within patients with COVID-19, where T-cell population is immensely variable, the single-cell profiling of T-cell subsets can be performed on a clinical cohort to understand insights of T-cell–mediated response and T-cell receptor repertoire dynamics. Detailed characterization of both CD4+ T cells and CD8+ memory T cells using single-cell omics can be helpful in patient categorization and plausible future protection. Understanding from the perspective of genes involved in defining the particular cell states during severe COVID-19 can also help us to elucidate the role of cellular heterogeneity in disease severity. Single-cell omics approach will help address the unresolved query concerning cellular heterogeneity that may assist us in preparing for the future as the SARS-CoV-2 is continuously evolving with acquired immune escape capabilities.

**Figure 4:**
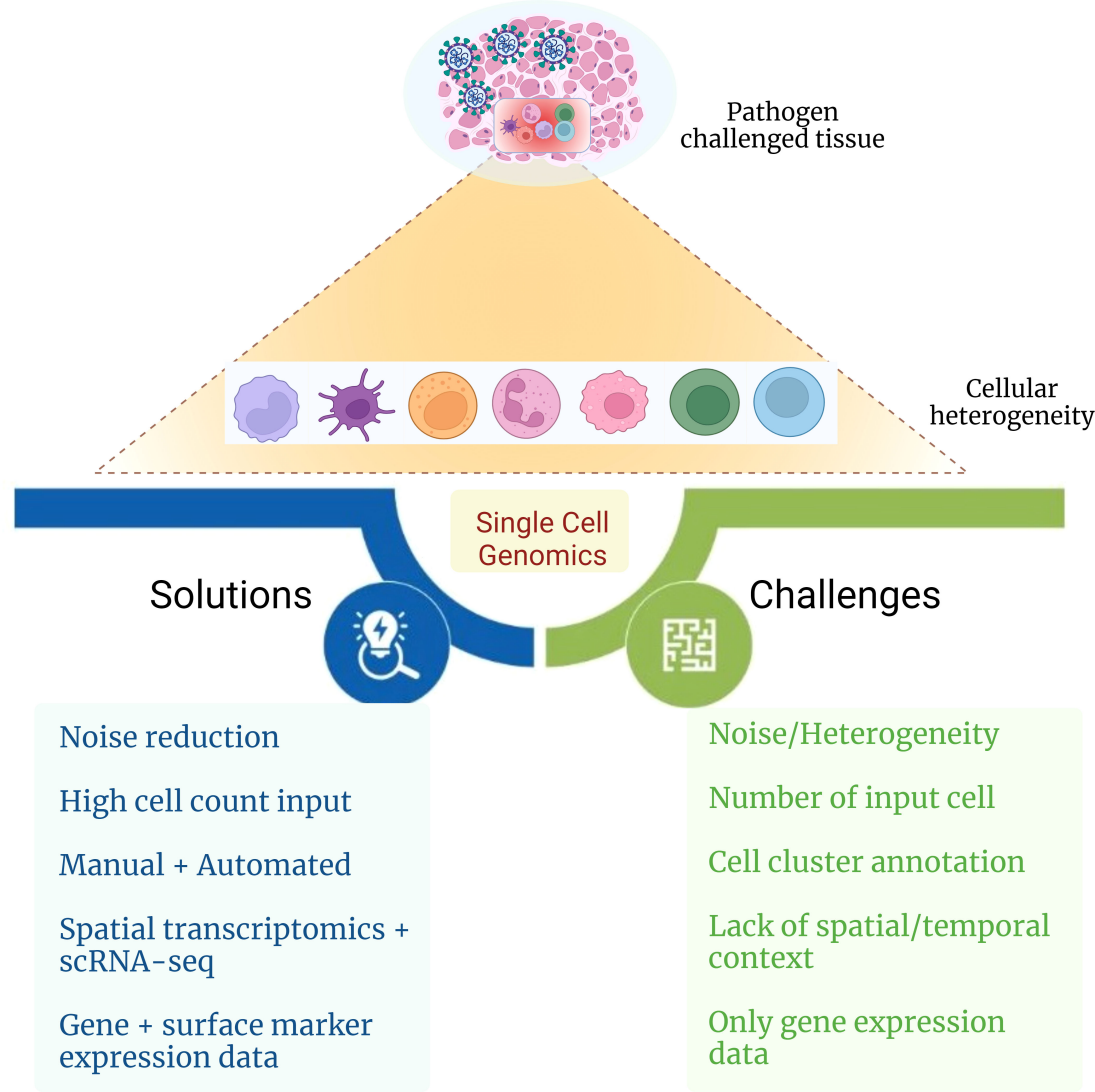
Challenges and possible solutions for delineating cellular heterogeneity. The figure captures challenges and possible solutions toward elucidating the role of cellular heterogeneity during infectious disease with the aid of single-cell genomics approach.

Understanding cellular heterogeneity in COVID-19 can be a cumbersome task as it comes with many challenges (**Figure 4**). The data generated from scRNA-seq are quite complex and have big volume, and multiple levels of quality check should be taken care of while arriving at the precise result. Often, the cell type identified can be misleading due to its probability of being a false result or noise. Hence, it is crucial to eliminate the noise in the data because each cell type and its expression contribute enormously to delineating patient heterogeneity. Even a low number of cells as input data can also cause a reduction in the discovery of rare cell types that usually are present in a low proportion. To avoid this, cell count as input should be above a threshold to capture even the low percentage of cell types. Generally, annotation of cell clusters is performed manually, which is labor-intensive that reduces the chances of achieving high accuracy in the results. With the help of multiple automated platforms and tools such as SCSA (a cell type annotation tool for single-cell RNA-seq data) and MACA (marker-based automatic cell-type annotation), heterogeneous cell populations may be captured efficiently within a patient (159, 160). Sometimes, the location of a particular cell type in a tissue can also be mistaken, which may lead to wrong interpretation of the spatial and temporal characteristics of the cell. Spatial transcriptomics, together with the scRNA-seq technique, can be performed, which is a high-throughput approach that can assist in providing the exact position with the expression of the cell from the tissue of interest. Gene expression may not provide enough clarity in detecting the granularity of heterogeneous cell populations. Therefore, it can be united with surface marker expression, further aiding elucidation of cellular heterogeneity. Altogether, the solutions toward resolving these challenges associated with single-cell omics technology in the future may help to develop a better understanding of the intricacies associated with cellular heterogeneity and provide substantial insights into the complexity behind COVID-19 disease.

## Author contributions

Author contribution KK has read the relevant literature, synthesized the findings, wrote the manuscript and made the figures. RP planned the study, wrote the manuscript, coordinated the manuscript outline, figures, conclusions and future challenges/opportunities. Both the authors contributed to the article, have read and approved the final submission.

## Funding

The study was supported by Bill and Melinda Gates Foundation (BMGF), INV-033578, Foundation for Innovative New Diagnostics (FIND), project code – GAP-0249, and AIDS Healthcare Foundation (AHF), project code CLP0043.

## Acknowledgments

KK acknowledges the fellowship from CSIR toward her salary support.

## Conflict of interest

The authors declare that the article was written in the absence of any commercial or financial relationships that could be construed as a potential conflict of interest.

## Publisher’s note

All claims expressed in this article are solely those of the authors and do not necessarily represent those of their affiliated organizations, or those of the publisher, the editors and the reviewers. Any product that may be evaluated in this article, or claim that may be made by its manufacturer, is not guaranteed or endorsed by the publisher.
